# A hybrid Cycle GAN-based lightweight road perception pipeline for road dataset generation for Urban mobility

**DOI:** 10.1371/journal.pone.0293978

**Published:** 2023-11-30

**Authors:** Balaji Ganesh Rajagopal, Manish Kumar, Abdulaziz H. Alshehri, Fayez Alanazi, Ahmed farouk Deifalla, Ahmed M. Yosri, Abdelhalim Azam

**Affiliations:** 1 Department of Computer Science and Engineering, SRM Institute of Science and Technology (SRMIST), Tiruchirappalli Campus, Tamil Nadu, India; 2 Department of Civil Engineering, SRM Institute of Science and Technology (SRMIST), Tiruchirappalli Campus, Tamil Nadu, India; 3 Department of Civil Engineering, College of Engineering, Najran University, Najran, Saudi Arabia; 4 Department of Civil Engineering, College of Engineering, Jouf University, Sakaka, Saudi Arabia; 5 Structural Engineering and Construction Management Department, Future University in Egypt, New Cairo, Egypt; 6 Public Works Engineering Department, Faculty of Engineering, Mansoura University, Mansoura, Egypt; Fudan University, CHINA

## Abstract

One of the major problems that cause continual trouble in deep learning networks is that training a large network requires massive labelled datasets. The preparation of a massive labelled dataset is a cumbersome task and requires lot of human interventions. This paper proposes a novel generator network ‘Sim2Real’ transfer is a recent and fast-developing field in machine learning used to bridge the gap between simulated and real data. Training with simulated datasets often converges due to its size but fails to generalize real-world applications. Simulated datasets can be used to train and test deep learning models, enables the development and evaluation of new algorithms and architectures. By simulating road dataset, researchers can generate large amounts of realistic road-traffic dataset that can be used to study and understand several problems such as vehicular object tracking and classification, traffic situation analysis etc. The main advantage of such a transfer algorithm is to use the abundance of a simulated dataset to generate huge realistic-looking datasets to solve data-intense tasks. This work presents a novel, robust sim2real algorithm that converts the labels of a semantic segmentation map to a realistic-looking street view using the Cityscapes dataset and aims to achieve robust urban mobility for smart cities. Further, the generalizability of the Cycle Generative Adversarial Network (CycleGAN) architecture was tested by using an origami robot dataset for sim2real transfer. We show that the results were found to be qualitatively satisfactory for different traffic analysis applications. In addition, road perception was done using a lightweight SVM pipeline and evaluated on the KITTI dataset. We have incorporated Cycle Consistency Loss and Identity Loss as the metrics to evaluate the performance of the proposed Cycle GAN model. We inferred that the proposed Cycle GAN model provides an Identity loss of less than 0.2 in both the Cityscapes dataset and KITTI datasets. Also, we understand that the super-pixel resolution has a good impact on the quantitative results of the proposed Cycle GAN models.

## 1. Introduction

Autonomous vehicles are an inevitable part of the upcoming decades due to their many benefits. Once a majority of the vehicles become self-driving, traffic can almost be reduced to zero, accidents can be heavily minimized, and navigation becomes very easy [1, 2]. With all the innumerable advantages, the development of autonomous vehicles also comes with major obstacles. The current technology faces the limitation of lack of a huge amount of labeled real datasets and the huge cost of computation [3–5]. For the development of robotic agents, simulation is a valuable method. Although simulation has long been used in robotics education and integrated robot software testing, there is a controversy in the scientific community about the ability to translate robotics skills learned in simulation to reality, a terminology known as Sim2Real transfer [6, 7]. Explicitly designing or learning intermediate representations that generalize between simulation data and real data is a good Sim2Real technique. Learning in simulation has the advantage of being faster than real-time, cheaper, safer, and more insightful (for example, by having ideal ground truth labels) than real-world experimentation. While it is possible to generate an infinite amount of labeled simulated dataset datasets theoretically, the generalization of the simulated dataset to the real world is poor. Also, the labeled datasets available are only in the order of 1000s images. However, this isn’t sufficient to satiate the thirst for the exponentially growing size of deep learning algorithms. Another obstacle that we face is the lack of low-cost processing power for performing the computations in the algorithm. While it is possible to use high-cost processors/GPUs to train offline models, the processors embedded in an autonomous vehicle must be lightweight in order to increase the speed and reduce the cost [8]. In this research work, we quantified the use of a sim2real-based offline deep learning model to generate datasets and a lightweight road perception pipeline for online computation. Traditional sensor systems, computer decision systems, and driving control systems are typically used in autonomous vehicles. The sensing system’s aim is to collect surrounding environmental information about the vehicle’s driving condition and provide data to the decision controller. Perception of roads and lanes is a significant aspect of any self-driving vehicle [9], enabling it to navigate autonomously. Computer Vision plays a critical role in enabling the vehicle to ’see’ what is around it and act accordingly [10].

One popular method is Generative Adversarial Networks (GANs), which consist of two neural networks: a generator and a discriminator. The generator produces synthetic data, while the discriminator attempts to distinguish the synthetic data from real data. The generator and discriminator are trained in an adversarial manner, with the generator trying to produce data that can fool the discriminator and the discriminator trying to identify the synthetic data correctly. GANs have been used to generate a wide range of data, including images, text, and speech.

Another method is Variational Autoencoders (VAEs), which consist of an encoder, a decoder, and a loss function. The encoder maps the input data to a latent space, the decoder maps the latent space back to the original data space, and the loss function measures the difference between the original and decoded data. VAEs have been used for image generation and data compression.

A third method is Autoregressive models, which predict each element of a sequence based on the previous elements. Examples of autoregressive models are PixelRNN and PixelCNN which have been used to generate natural images.

A fourth method is Flow-based models, a class of generative models that can transform a simple random noise into a complex data distribution. Examples of flow-based models are Real NVP, Glow, and Normalizing Flows. These flow-based models have been used to develop natural images based on the distribution of image pixels.

In summary, GANs, VAEs, Autoregressive models, and Flow-based models are all popular methods for generating simulated datasets for deep learning. Each method has its own unique characteristics and is suited to different types of data and tasks. GANs and VAEs are more suited to image generation, while Autoregressive models and Flow-based models are more suited to sequence generation tasks such as text and speech. GANs are popularly used for Image Generation tasks, including generating simulated datasets for road traffic perception pipelines. Section 2 describes the various research works related to GANs and their different types.

In Section 2, we provide a review of existing methods in the field of sim2real, road perception and SuperPixel extraction. Section 3 shows the methodology adopted to model the required algorithms. Section 4, presents the results of the sim2real transfer using our adopted method and the perception of the road using SuperPixel classification. We illustrate the qualitative results of both tasks in the form of output images. In addition, we also show the reproducibility of the results using a completely different dataset. The major contributions in this work are: (1) Developing a robust sim2real algorithm to both utilize the power of simulations and obtain a labeled realistic-looking dataset that resembles the practical data; and (2) A lightweight machine learning pipeline for road perception [11, 12].

## 2. Related work

### 2.1 Why Cycle GANs for generations of simulated images?

Cycle GANs are class of Generative Adversarial Network (GAN) specifically designed for image-to-image translation tasks. They are an extension of the original GAN architecture and have several unique features, such as (i) Cycle GANs do not require paired training data, i.e., they can translate images from one domain to another without having corresponding images in the other domain. (ii) Cycle GANs use a cycle consistency loss. This loss measures the difference between the original and translated images after being translated back to the original domain. (iii) Cycle GANs have two generators and two discriminators. Each generator learns to translate images from one domain to another and each discriminator is trained to identify the translated images. (iv) Cycle GANs are able to generate high-quality images that are often difficult to distinguish from real images. Cycle GANs can be trained on small datasets and still produce good results. (v) Cycle GANs have been used for a variety of image-to-image translation tasks, such as style transfer, super-resolution, and object transfiguration.

### 2.2 Sim2Real

Sim2Real transfer is the technique of adapting models or policies trained in simulation to work in the real world. There are several ways to perform Sim2Real transfer, including fine-tuning, domain adaptation, adversarial training, and hybrid simulation. The robotics community has used simulations and modeling systems from the initial stages of development. Although simulation has long been used for research and prototyping, the robotics community has only recently attempted to translate simulation-based behaviors to the real world (this process is usually referred to as Sim2Real). Even though we have made decent progress in translating simulation-based behavior to reality, most proposed solutions still struggle to reliably address the reality gap without substantial fine-tuning and learning in the real environment. When visual sensors like cameras are involved, things worsen, as simulating actual sensors widens the reality gap even further [13]. While robotic simulations are easily available and accessible, this research subject invites contributions that will enable real complex systems like autonomous vehicles with real-world sensors to learn with limited physical data. The adversarial training used in this research work consists of both generator and discriminator, where a discriminator is trained to distinguish between real and simulated data, and the generator is trained to produce simulated data that is indistinguishable from real data. This research work aims to converge multiple methods from literatures into single pipeline which will enable the road perception pipeline to learn diversified road environments using simulations rather than constrained road environments.

### 2.3 Road perception

The capacity of an autonomous device to gather information and derive specific knowledge from its surroundings is referred to as perception. Environmental interpretation is the process of gaining a qualitative awareness of the environment, such as locating hazards, detecting road signs and markings, and categorizing data according to its conceptual sense. The primary visual cues for human driving are road color, texture, and markings. Since autonomous vehicles are supposed to share the road with human drivers, they should also extract and analyze these visual cues. In recent years, there has been considerable progress in detecting roads and lanes using various sensors like monocular cameras, stereo cameras, and LiDARs. While it is true that, more the data, the more we have to analyze, it is not always possible when the data availability is bare minimum. In recent years, enterprises like Tesla Motors [14] have developed algorithms that rely solely on monocular vision (in addition to radars) to automate self-driving completely. The reason is that when human drivers can skillfully perceive the road conditions just with vision, it should be possible for an artificially intelligent system to do the same. This helps cut costs as LiDARs are expensive and speed up the algorithm.

### 2.4 SuperPixel extraction

A single image contains tens of thousands of pixels, and processing such a huge amount of data by accessing data at the pixel level is highly computationally expensive. SuperPixels are an effective solution to solve this issue. A SuperPixel is an image patch containing a set of pixels sharing similar features. Super-pixel extraction is useful in several computer vision tasks, such as object detection, semantic segmentation, and image restoration. Super-pixels are smaller than traditional image segments and more semantically meaningful, making them useful for tasks requiring a higher level of detail. SuperPixels are also effective for semantic analysis of the image, as individual pixel data is meaningless. There are numerous ways to extract SuperPixels from a given image.

Deep learning is used to extract the super-pixel, by training a neural network to predict super-pixel labels for each pixel in an image. Deep learning-based super-pixel extraction methods are generally based on fully convolutional neural networks (FCNs), which are able to process images of any size. But when there are constraints in the availability of the data, deep learning-based super-pixel extraction methods are not useful. Also, Deep learning-based super-pixel extraction methods are not useful in case of homogeneity of image datasets. In this research work, the road image dataset is used, and it is difficult to generalize the network for road images dataset for super-pixel extraction.

Hence, We have used the simple linear iterative clustering method (SLIC) to extract the SuperPixels [15, 16]. SLIC algorithm is based on K-means clustering, and it uses a compactness constraint to form compact and spatially regular super-pixels. It is a novel algorithm that efficiently generates compact, nearly uniform SuperPixels by clustering pixels in a combined five-dimensional color and image plane space. SLIC generates SuperPixels at a lower computational cost than four state-of-the-art approaches, as calculated by boundary recall and under-segmentation error. This algorithm works on the principle of gradient ascent algorithm.

Starting from an initial rough clustering, during each iteration, gradient ascent methods refine the clusters from the previous iteration to obtain better segmentation until convergence. Another important use of SLIC in this application is its high computational speed.

**Fig 1:**
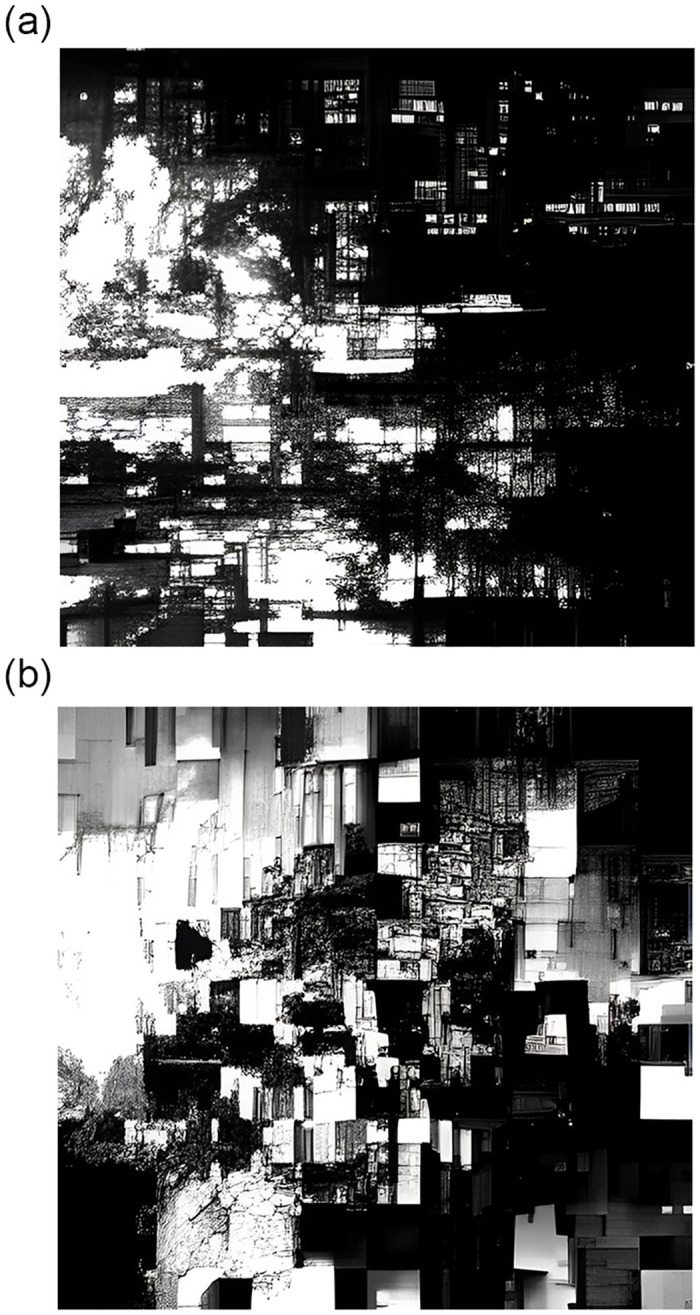
Results of Super-pixel extraction between SLIC(Left) and k-means(right) algorithms.

SLIC performs exceptionally well in cases where the number of SuperPixels involved starts increasing. In such a case, the time taken for the k-means clustering algorithm increases exponentially while SLIC performs relatively faster, making it suitable for real-time images. The key to speed up the SLIC algorithm is limiting the size of the search region hence significantly reducing the number of distance calculations and results in a significant speed advantage over conventional k-means clustering where each pixel must be compared with all cluster centers. The Fig 1 shows the difference between super-pixel clustering for SLIC and k-means for the same seed value of the random distribution of gray level (0–255).

### 2.5 Synthetic road image generation

Various research litertures which used CycleGAN architecture for generation of Synthetic Image dataset is presented in this section, highlighting the contributions made by each work and the identified research gap is presented at the end of this section.

Zhu et al., [17], introduced the CycleGAN, a GAN architecture for image-to-image translation using unpaired data and cycle consistency loss. The authors showed that the method is able to generate high-quality images that are often difficult to distinguish from real images.

Saleh et al., [18] proposed a method for generating synthetic roadside images using Cycle GANs. The authors used a large-scale dataset of real-world urban images and used the Cycle GANs to generate synthetic images that were similar in terms of resolution, texture, and appearance. They showed that their method was able to produce high-quality images suitable for use in scene understanding tasks.

Park et. al [19] proposed a method for generating synthetic roadside images using Cycle GANs. The authors used a dataset of real-world urban images and used the Cycle GANs to generate synthetic images that were similar in terms of resolution, texture, and appearance. They showed that their method was able to produce high-quality images suitable for use in scene understanding tasks. They also propose a framework that combines the generated synthetic images with real-world images to improve the performance of scene understanding tasks.

"Wang et al. [20] proposed a method for generating synthetic road side images using Cycle GANs. The authors used a dataset of real-world urban images and used the Cycle GANs to generate synthetic images that were similar in terms of resolution, texture, and appearance. They showed that their method was able to produce high-quality images suitable for use in scene understanding tasks. They also proposed a method for creating realistic lighting conditions for the synthetic images.

Little [21] proposed a method for synthesizing images of scenes using CycleGANs for use in a scene understanding system. The method is able to generate realistic images of scenes with a wide range of objects and is suitable for tasks such as data augmentation and image synthesis.

Pang et al [22] proposed a method for image-to-image translation using multi-level consistency, which includes both pixel-level consistency and semantic-level consistency. The method uses cycle consistency loss to ensure that the generated images are faithful to the original images and is able to handle multiple modalities such as images and text.

Yag et. Al. [23] presents various hybrid deep learning models for early detection of plant diseases. This work used two generators and discriminators networks to detect the abnormality in the plants leaves.

Karasu and Altan [24] proposed a novel Region-based Convolutional Neural Network (R-CNN) from deep learning approaches with Multi-Layer Perceptron (MLP) and Support Vector Machine (SVM) from machine learning approaches in order to distinguish agricultural crops from weeds. This paper proposed the significance of introducing SVM for distinguishing the crops and weeds.

Pix2PixHD is a generative model, introduced in [25]. The model used a conditional GAN to generate realistic images from a semantic map with improvements such as multi-scale generator architecture, a feature matching loss, and a discriminator that operates on a global and local scale. A novel network ‘StarGAN v2’ for image synthesis was proposed in [26] used "style codes" in the generator network to enable diverse and flexible image synthesis across multiple domains with high-quality image synthesis, improved stability, and better generalization capabilities. The work in [27] used AttGAN model for makeup transfer which used a generative model that learns to generate diverse images for a given input image. The work used makeup style code and optimized it through an adaptive loss function. The paper [28] used Semantic Editing and Semantic Pyramid (SP) with Attentive Normalization model for image generation. This SP module consists of a series of semantic feature maps with increasing levels of abstraction, and are used to control the generation of different image regions. A novel approach for neural style transfer based on the MSG-Net (Multi-Scale Gradient Network) model is proposed in [29], which introduced a new "Gradient Domain Fusion" technique that uses multi-scale gradients to transfer the style of one image onto another. This approach improved the visual quality and content preservation of stylized images by better incorporating the texture information of the style image. The works in [30] proposed a model that combines the SPADE (SPatially ADaptive (DE)normalization) technique with a sketch-to-image translation network to generate high-quality photos from sketches. The proposed model takes into account both the sketch information and the semantic information of the target photo to produce realistic images. A novel regularization technique that reduces artifacts and improves the overall visual quality is proposed in [31]. This work highlighted the potential of StyleGAN2 for high-quality image generation applications, and provides insights into the underlying mechanisms of generative models. The results of these methods with the proposed Cycle GAN is presented in section 3.

In summary, these works proposed the use of Cycle GANs for generating synthetic road side image datasets. All the works [17–31] used real-world urban images as the source dataset, and used the Cycle GANs to generate synthetic images that were similar in terms of resolution, texture, and appearance. They all showed that their method was able to produce high-quality images suitable for use in scene understanding tasks, and some of them proposed additional features such as realistic lighting conditions, and combining synthetic and real-world images to improve the performance of scene understanding tasks. Still the researchers find it difficult in adapting CycleGANs to unseen domains, possibly through domain adaptation techniques or meta-learning approaches. Also, the Environmental conditions, lighting, and viewpoint changes can significantly affect the quality of synthesized images. In such cases, the number of generator and discriminator networks can be optimized with hyper parameters, which will also reduce the vanishing gradients problem. The mode collapse of the generator network can also be addressed with the help of discriminative classifiers like Support Vector Machine.

### 2.6 Support vector machine

A Support Vector Machine (SVM) [32] is a discriminative classifier formally defined by a separating hyperplane. In other words, given labelled training data, the algorithm outputs an optimal hyperplane that categorizes new examples. In two-dimensional space, this hyperplane is a line dividing a plane into two parts wherein each class lay on either side. In this case, we used a 10-dimensional space for binary classification. The important parameters of an SVM classifier are the kernel type, C, gamma (for rbf). The rbf kernel is chosen sine the features aren’t linearly separable, hence transformed into a new plane before classification. The gamma parameter defines how far the influence of a single training example reaches, with low values meaning ’far’ and high values meaning ’close’. We have used a low value since, with such a huge dataset and the noisy nature due to textural and color similarity of pavements, a high value of gamma would result in overfitting. The C parameter trades off the correct classification of training examples against maximization of the decision function’s margin. A smaller value of C has been used in this case as it encourages a larger margin on the cost of misclassifying a few points. This is desired since having a larger margin ensures the generalization of the model while ignoring few data due to noises.

In the context of Cycle GANs, SVMs can be used as a classifier in the discriminator network of the GAN. An SVM classifier can be used as the discriminator in a Cycle GAN to classify the generated images as either real or synthetic. The SVM classifier is trained on a dataset of real images and synthetic images generated by the generator network. It can then be used to evaluate the quality of the synthetic images generated by the generator network by determining how well they are classified as real images by the SVM classifier.

Additionally, SVMs are also used to improve the performance of the generator network by providing it with feedback on the quality of the synthetic images it is generating. This can help the generator network to learn to generate synthetic images that are more similar to the original images and therefore more likely to be classified as real images by the SVM classifierSVMs are used in the proposed Cycle GANs to improve the performance of the generator network by providing it with feedback on the quality of the synthetic images it is generating and SVMs are also used to classify the generated images as real or fake.

## 3. Materials and methods

### 3.1 Dataset preparation for Sim2Real transfer

**Fig 2:**
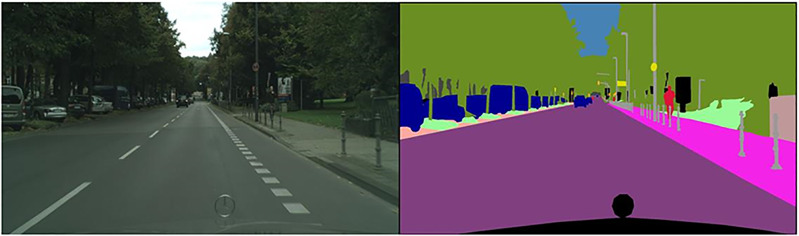
A sample image from the cityscapes dataset showing an image pair of the street view and a semantic segmentation map shown as different color labels.

The ideal dataset, in this case, would be an unpaired set of simulated and real images that can be used for the sim2real mapping. However, working on a good enough simulator to generate a huge dataset as per our requirement can itself be a separate project. Instead, we use the Cityscapes dataset [33–35], which contains the street view and the sematic segmentation labels. This dataset has been obtained from 50 different cities all around Europe and contains pixel-level annotated data for 5000 images. The labels here can substitute the simulated data as they plain and contain minimal features. While the dataset contains paired set of images, this method works for an unpaired dataset as well. A sample image of the Cityscapes dataset is shown below as Fig 2. The standard data preparation techniques like resizing and normalization are done before starting the training. Due to the big size of the model, the batch size was restricted to one.

### 3.2 Sim2Real transfer using CycleGAN

**Fig 3:**
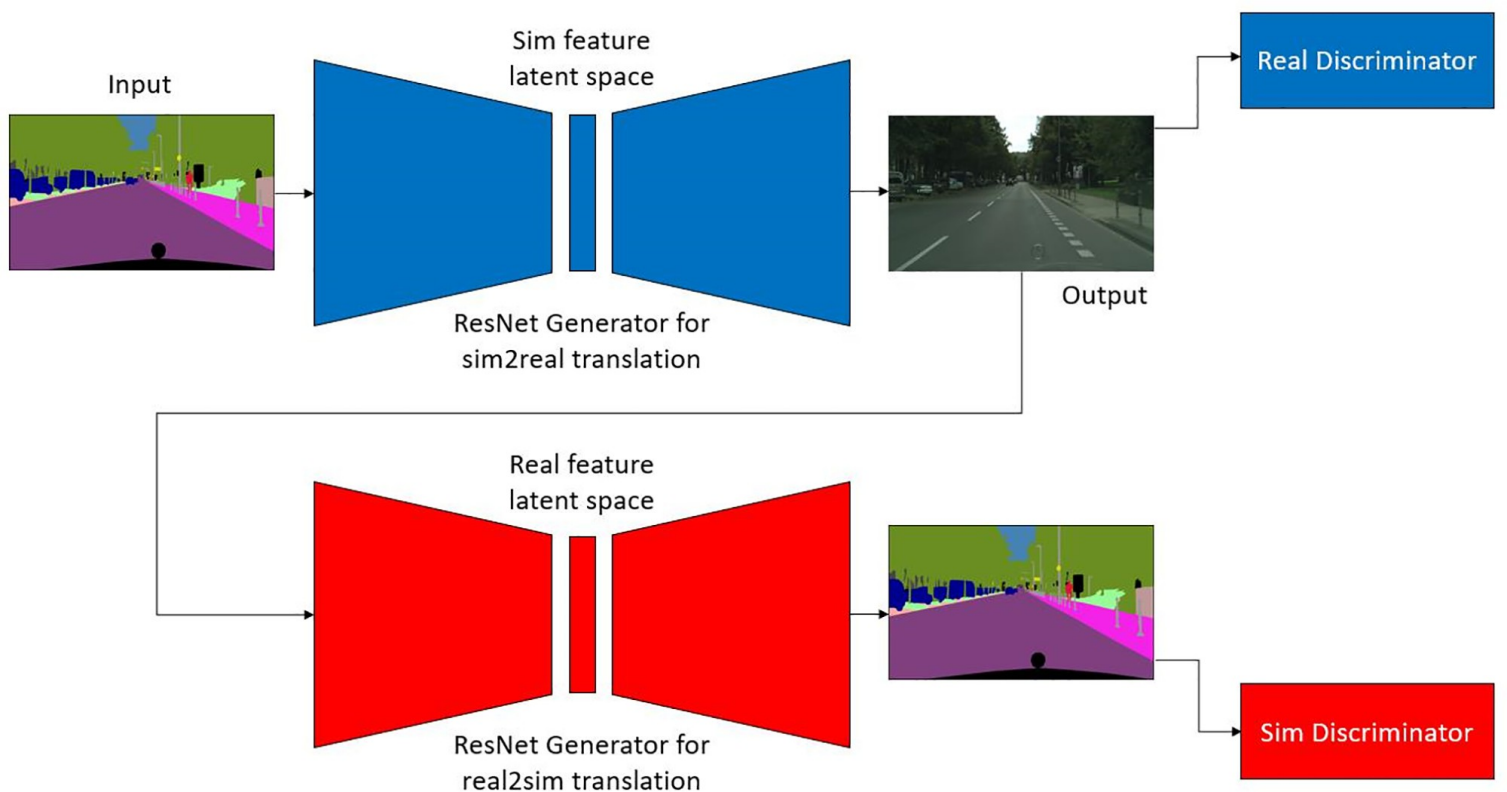
Block representation of the sim2real CycleGAN using a ResNet generator and a patch discriminator (sim2real shown in blue and real2sim shown in red).

CycleGAN is a type of generative adversarial network that works on the principle of minimizing the cycle consistency loss [17]. CycleGAN is an architecture introduced in 2017 designed specifically to perform image-to-image translation on unpaired sets of images. There are four networks working together simultaneously during each iteration step in a CycleGAN. They are namely the generator and discriminators for the sim2real and real2sim transfers. The generator network is a ResNet-based image-to-image translation network. The discriminator uses image patches from the generator output image as inputs to decide the type of image (sim or real). The complete working of the proposed CycleGAN is shown in Fig 3. Apart from the cycle consistency loss, we also use the identity loss to ensure that the generator networks do no changes to the image of the same type. For example, if a real image is passed into the sim2real network the output should be the same input image without any alterations. This helps the generator networks just to learn the translation between both the types and not perform other unnecessary changes. This is particularly useful to keep the features like the positions of different features in the images like lanes, vehicles and humans to remain same. One major advantage of this method is that it is completely unsupervised. While we used manually annotated dataset in our project, this is not mandatory. The complete non-requirement of supervision in this method makes it easier to scale up the model size as manual work decreases by a significant amount. The training was performed until the discriminator losses were approximately equal to 0.5. This shows that the output quality is good enough to saturate the discriminator performance, as 0.5 means both the classes can have equal probability and the discriminator more likely was choosing at random. The training was performed for 35 epochs till both the discriminators were almost saturated. In general, it was observed that the generator loss converged quicker. The weight to cycle consistency loss with respect to identity loss was kept as 10 to prioritize sim2real and real2sim are inverse functions of each other. Adam was used as an optimizer for the loss functions during training. The hyperparameters used in this work are as follows:

**Learning Rate = 0.0002**,
**Number of Generator and Discriminator Layers = 2 layers each**
**Number of Filters = 64, each in Generator and Discriminator**
**Optimizer = Adam**
**Mini-Batch Size = 16**
**Number of Training Epochs = 35**
**Weights to Cycle consistency loss = 10**

These hyperparameters are set on random and we found that the lower the learning rate and reduced weights to cycle consistency loss improves the performance of the proposed cycle GAN for sim2real transfer. Since both the discriminators are saturated after 35 epochs, the training was stopped.

### 3.3 Dataset preparation for road perception

The labelled dataset from KITTI Vision Benchmark Suite [36] with 289 images containing 95 urban marked roads, 96 urban multiple marked roads and 98 urban unmarked images. The dataset also has 290 test images. Features like color aren’t fully sufficient in all the cases. Hence texture and other features of the road help in classifying the road from other similar parts of the image like pavements. A total of approximately 500 SuperPixels were extracted from each labelled image and 12 features were extracted per SuperPixel.

- Position (X, Y coordinates of the center) (2)
- Color (R, G, B average) (3)
- Shape (Perimeter and Area) (2)
- Contrast, Dissimilarity, Homogeneity textures (3)

**Fig 4:**
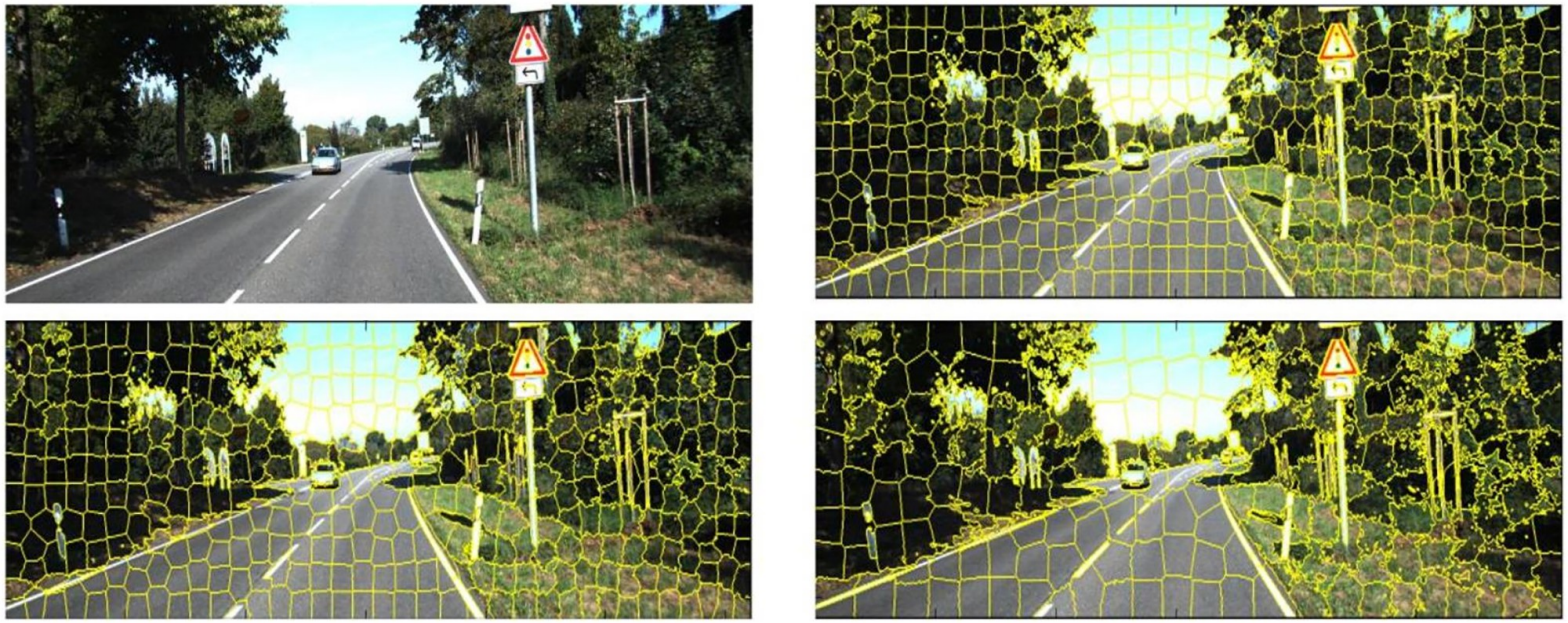
A sample image from the KITTI dataset, along with a different number of SuperPixel extracted, decreasing in the order of top right, bottom left, and bottom right.

The dataset comprising the extracted SuperPixel features is 2-dimensional with a size 140,000x11. Table 1 shows a sample dataset that has the features of few SuperPixels. Fig 4 shows a sample image from the KITTI dataset and image after SuperPixel extraction.

**Table 1:** Sample dataset after feature extraction used for SuperPixel classification.

X avg	Y avg	R avg	G avg	B avg	Peri.	Area	Contr.	Dis.	Hom.	Class
171.78	169.78	77.262	84.713	90.399	3029.7	32.794	0.0725	0.0271	0.1335	1
232.76	171.49	29.574	40.170	26.535	67.456	6.0245	0.1688	0.0512	0.4919	0
146.53	178.26	108.59	108.75	107.22	415.61	13.960	0.1087	0.0294	0.8598	1
6.7611	6.2388	241.21	247.39	251.36	2941.5	25.209	0.6094	0.5829	0.3771	0

### 3.4 Road perception using support vector machine

In the proposed road perception pipeline, most of the task is done after successfully extracting the features. To reduce the burden on system due to the already running computationally intensive tasks, a support vector machine is used to map the features to the output class of either 1 or 0 based on whether the particular SuperPixel belongs to the road or not. Support vector machine is apt here as it not only makes the algorithm computationally inexpensive and fast but also because of the simple mapping from features to a binary class since most of the work is done in the feature extraction.

## 4. Results

### 4.1 Formulating the GAN loss functions

Three different types of loss functions are considered in the proposed method, which, when minimized optimally, will give satisfactory results. The first loss under consideration is the different GAN losses of the generator and discriminator networks of the sim and real networks. We will have the GAN loss as a sum of the four different losses. The second type of loss is the cycle consistency loss which helps with the unpaired image translation. As shown in Fig 3, a label image is passed into the sim2real network, the output of this is passed into the real2sim network and this output is compared with the initial label image using L1 loss function. The goal of this loss function is to ensure that the real2sim network works inversely with respect to the sim2real network and vice versa. The last type of loss is identity loss, where a label image is passed into real2sim. This network should not make any change to the image and the output should be ’identical’, thus called the identity loss. The same applies to using street images as input to sim2real network.

### 4.2 Assessing the network performance

**Fig 5:**
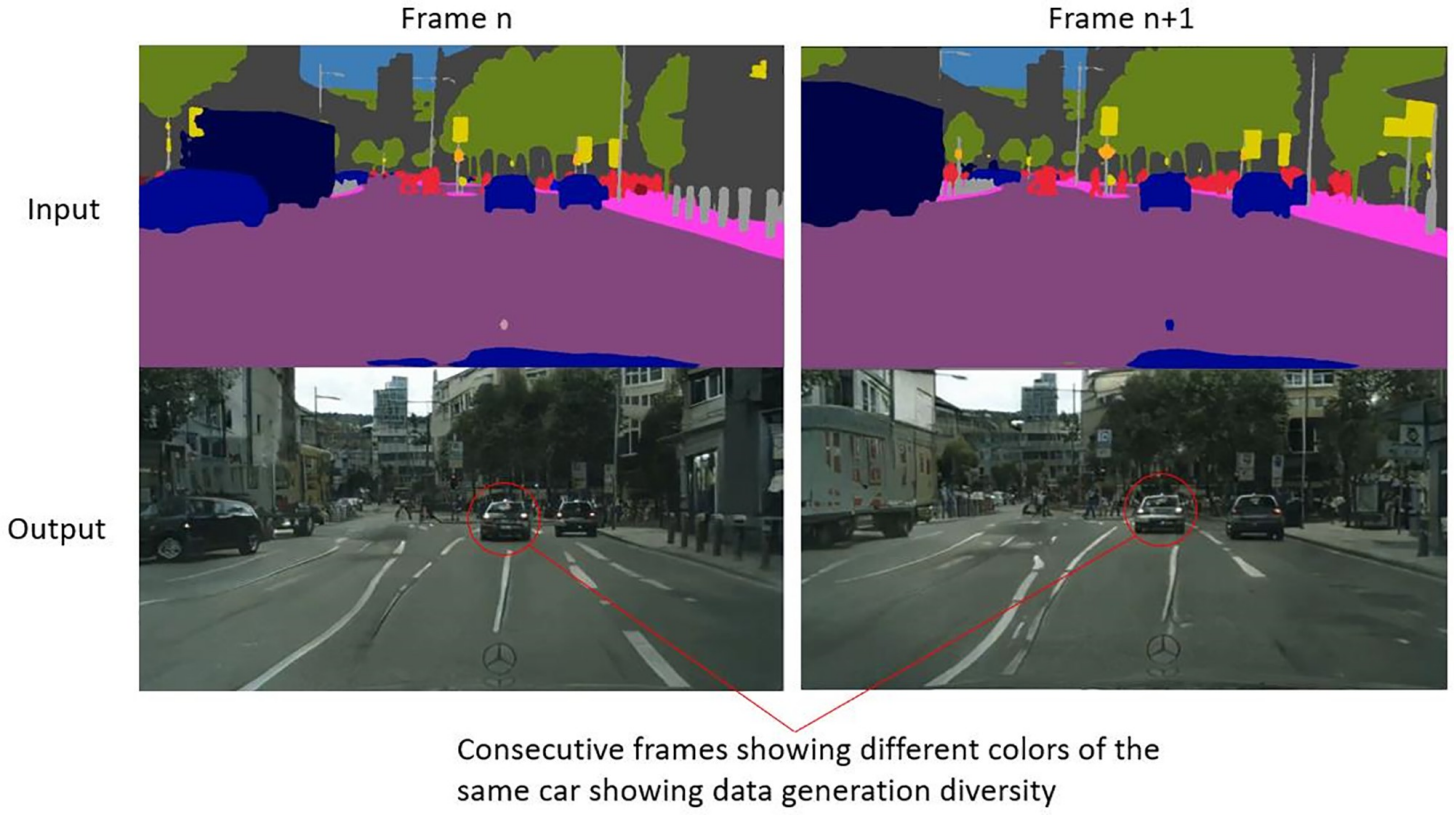
A qualitative representation of the results of the trained sim2real transfer algorithm.

The sim2real network translates an image with very little content to an image with complex structures such as buildings, trees, roads and cars. All this content is learned from the dataset and stored in the latent spaces of the generators. Thus, it is not reasonable to compare the output of the sim2real network with the ground truth in a quantitative nature. Although the quantitative properties of the image can’t be compared directly, the structure of the image is maintained. This is also advantageous as this can lead to different properties such as new car color, road lanes, textures etc. A qualitative comparison can be seen in Fig 5, showing sample images between labels, generated street view and ground truth. The variation can be clearly seen in the colors and textures of the cars and roads. This diversity can be really helpful to generate a robust dataset for training neural networks for autonomous vehicles [37]. Another advantage is the possibility to theoretically generate as much real-looking data as we want with semantic labels. Generating random labels is an easy task. Using these labels and the sim2real network, we can theoretically generate infinite corresponding street view images. This type of data augmentation can prove highly useful in the field of autonomous driving.

### 4.3 Quantitative analysis of the proposed Cycle GAN

Quantitative comparison of Cycle GAN, which is used to generate simulated dataset can be done using a variety of metrics such as Inception Score, Fréchet Inception Distance (FID), Structural Similarity Index (SSIM), Cycle consistency loss.

Inception Score (IS): This metric measures the quality and diversity of the generated images by evaluating the performance of a pre-trained classifier on the generated images. The higher the IS, the better the generated images are considered to be.

Fréchet Inception Distance (FID): This metric measures the distance between the feature representations of real and generated images in the feature space of a pre-trained classifier. The lower the FID, the more similar the real and generated images are considered to be.

Structural Similarity Index (SSIM): This metric measures the structural similarity between the original and generated images, taking into account factors such as luminance, contrast, and structure. A higher SSIM indicates that the generated images are more similar to the original images.

Cycle consistency loss: To ensure that the generated images are faithful to the original images, Cycle GANs use a cycle consistency loss. This loss measures the difference between the original image and the translated image after it has been translated back to the original domain.

Identity loss is a term used in Cycle GANs to refer to the loss that ensures that the generator network is able to maintain the content and structure of the original images when translating them to another domain. It is a way to ensure that the generator is able to preserve the information of the original images when translating them. It is called "identity loss" because it is used to keep the identity or content of the original image intact during the translation process. The idea is to minimize the difference between the original images and the images that are translated and then translated back again.

In this work, we have used both Identity loss and Cycle consistency loss to ensure that the generated images are faithful to the original images in the cityscape paired Image datasets, KITTI datasets and the results are presented in Table 2.

**Table 2:** Performance metrics of our Sim2Real transfer model in different datasets.

Datasets	Identity Loss		Cycle Consistency Loss	
	With Super-pixel Classification	With-out Super-pixel Classification	With Super-pixel Classification	With-out Super-pixel Classification
Cityscapes paired Image datasets	0.21	0.35	0.38	0.42
KITTI dataset	0.19	0.28	0.25	0.31

### 4.4. Results of the proposed Cycle GAN with SOTA models

The results of the proposed CycleGAN for generating sim2real dataset has been compared with other state-of-the-art deep learning models. Different deep learning models use variety of datasets and parameters to evaluate the performance of the Image Generation models. Among various performance metrics, Fréchet Inception Distance (FID), Inception Score (IS), Structural Similarity (SSIM), are the most widely used metric in the research papers referenced in section 2.

Fréchet Inception Distance (FID): FID measures the distance between the feature representations of real and generated images and FID is calculated using Eq (4.1.)

$$FID={\left|\left|\mu_{r}-\;\mu_{g}\right|\right|}^{2}+\;Tr\left(S_{r}+\;S_{g}-\;2{\left(S_{r}*S_{g}\right)}^{\frac{1}{2}}\right)$$ (4.1.)

**where**,

FID is the Fréchet Inception Distance

||μ_*r*_ − μ_*g*_||^2^ denotes the squared Euclidean distance between two vectors

μ_*r*_ and μ_*g*_ are the mean vectors of the real and generated data, respectively

*S*_*r*_ and *S*_*g*_ are the covariance matrices of the real and generated data, respectively

*Tr* denotes the trace operator

* denotes the element-wise product (Hadamard product)

Inception Score (IS): IS measures the diversity and quality of the generated images. Inception Score is calculated using Eq (4.2)

$$IS=\text{exp}\left(\frac{1}{N}\;\left(\sum_{i=1}^{N}\left(KL\;\left(\frac{p\left(y|x_{i}\right)}{p\left(y\right)}\right)\right)\right)\right)$$ (4.2)

*where*,

*N is the number of generated images*

*p*(*y*│*x*_*i*_) *is the conditional class distribution of Inception v*3 *network given an image*

*p*(*y*) *is the marginal class distribution estimated by averaging* \*the conditional distribution over all the generated images*

{*KL*} *denotes the Kullback*–*Leibler divergence*.

Structural Similarity (SSIM): SSIM measures the structural similarity between two images. SSIM is calculated using Eq (4.3)

$$SSIM\left(x,y\right)=\frac{\left(2\;\mu_{x}\mu_{y}+\;c_{1}\right)\left(2\sigma_{\left\{xy\right\}}+\;c_{2}\right)}{\left(\mu_{x}^{2}+\;\mu_{y}^{2}+\;c_{1}\right)\left(\sigma_{x}^{2}+\;\sigma_{y}^{2}+\;c_{2}\right)}$$ (4.3)

Here, x and y are the generated and reference images, respectively, σ_x_ and μ_y_ are the mean intensities of the images, σ_x_ and σ_y_ are the standard deviations of the images, σ_xy_ is the cross-covariance of the images, and c_1_ and c_2_ are constants to avoid instability when the denominator is close to zero.

LPIPS (Learned Perceptual Image Patch Similarity) is a metric used to measure the similarity between two images based on the perceptual information learned from a pre-trained deep neural network. LPIPS is frequently used as a metric for evaluating the performance of generative adversarial networks (GANs) and variational autoencoders (VAEs), and for comparing the quality of generated images to real images. The LPIPS metric measures the distance between the feature representations of the two images, where a smaller distance indicates a higher perceptual similarity between the images.

$$LPIPS\left(x,y\right)=\frac{1}{n}\sum_{i=1}^{n}||\phi_{i}\left(x\right)-\phi_{i}\left(y\right)|{|}^{2}$$ (4.4)

where,

*n* is the number of patches extracted from each image

*ϕ*_*i*_ is the ith patch of the image to be compared

The performance comparison of the different SOTA deep generative models with respect to the proposed CycleGAN model for generation of real dataset for autonomous vehicles is presented in Table 3. ‘NA’ in Table 3 indicates that the metric was not reported in the respective paper. The lower values for FID and LPIPS indicate better quality of the generated images, while a higher IS, SSIM indicates better diversity of generated images.

**Table 3:** Performance comparison of the proposed CycleGAN with other SOTA deep generation models.

Model	Works carried out	Dataset used	Performance Metrics			
			FID (Lower is better)	IS (Higher is better)	SSIM (Higher is better)	LPIPS (lower is better)
**Pix2PixHD**	**High-Resolution Image Synthesis and Semantic Manipulation with Conditional GANs**	**Cityscapes**	**27.4**	**4.68**	**NA**	**0.873**
**StarGAN v2**	**StarGAN v2: Diverse Image Synthesis for Multiple Domains**	**Sketch & Cartoon**	**13.11**	**52.21**	**NA**	**0.332**
**AttGAN**	**AttGAN: Facial Attribute Editing by only Changing What You Want**	**CelebA**	**24.34**	**NA**	**0.810**	**0.409**
**SEAN**	**Semantic Pyramid for Image Generation with SEAN**	**PASCAL VOC**	**12.67**	**24.63**	**NA**	**NA**
**MSG-Net**	**Neural Style Transfer via Multi-Scale Gradient Domain Fusion**	**Places2**	**26.52**	**NA**	**0.813**	**0.191**
**StyleGAN**	**StyleGAN2: Analyzing and Improving the Image Quality of StyleGAN**	**FFHQ (Flickr-Faces-HQ)**	**18.58**	**9.83**	**NA**	**NA**
**Proposed CycleGAN for Sim2Real**	**Generation of Simulated dataset for Autonomous Vehicles**	**KITTI Dataset**	**11.56**	**53.89**	**0.801**	**0.108**

### 4.5 Additional results on unpaired dataset

**Fig 6:**
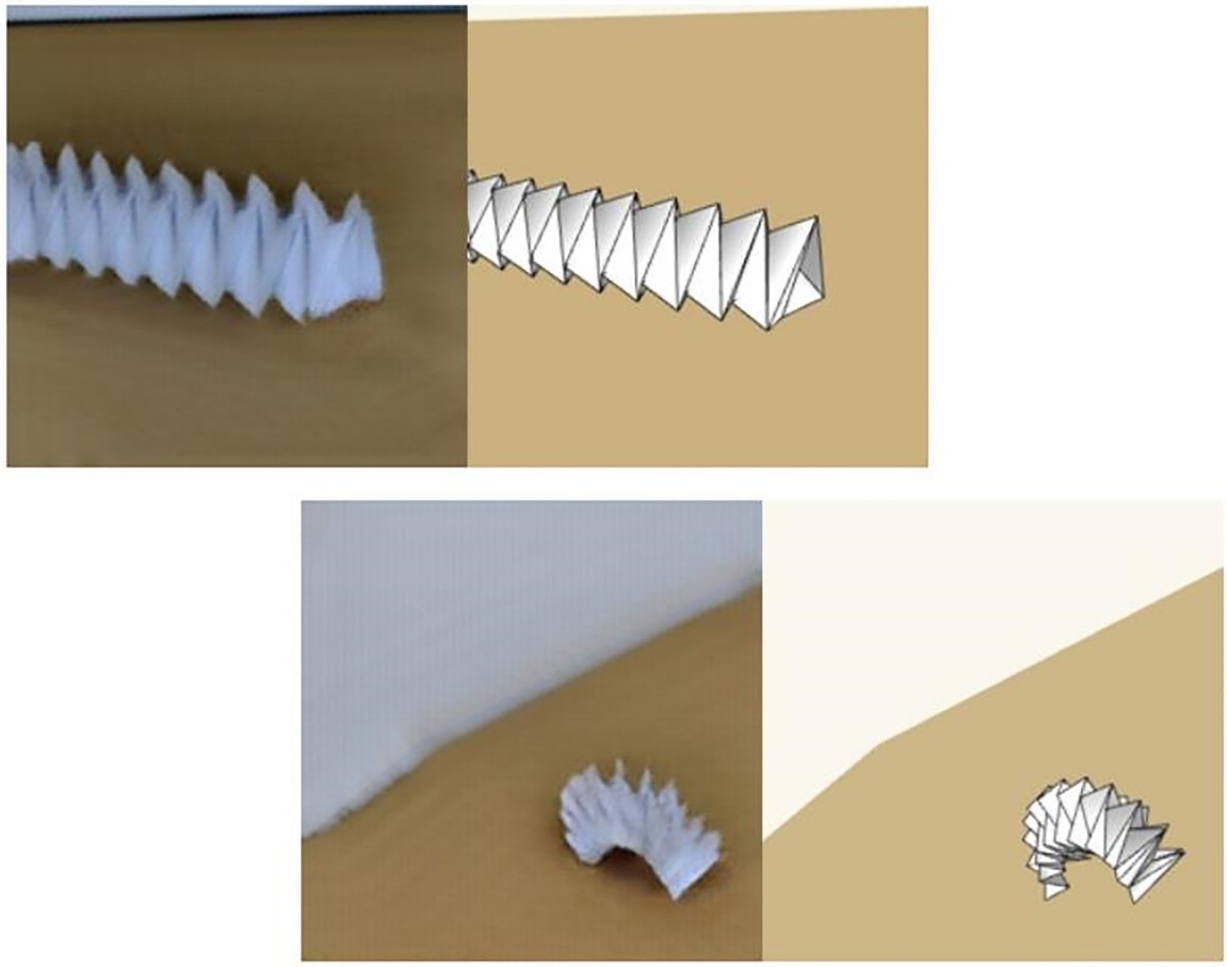
Shape, size and pose of the robot remaining the same while additional image complexity is added to make it look more realistic.

While the main aim of the proposed project is to use CycleGAN for generating sim2real datasets for autonomous vehicles, it is also important to test the effectiveness of the method using other datasets. Another reason to use additional datasets is also to check the capability in unpaired datasets as the Cityscapes dataset provides paired images. An origami robot simulation [38] is used to generate the required simulated dataset. A handmade origami robot is used to generate a real dataset. This sim2real transfer has similar applications to the above described task. The simulation can be used to generate any size dataset, but the features of the image are plain and not enough to use for tasks like pose estimation or tracking. These tasks might even sometimes converge with a few epochs when trained using a simulated dataset due to the lack of complexity in the image. This also poses the issue of using the trained model on real conditions since, that is the end goal. The same sim2real network proposed is used to add complexities that can be found in real images of the robots and superpose it on the simulated image structure, thus giving us control over complexity and other features such as shape, size and pose. The results of the origami robot sim2real transfer are depicted in Fig 6, where it can be seen that the generated image resembles a real image while maintaining the originality from the simulated image.

### 4.6 Perception of roads

The SVM algorithm was used to identify the boundary vectors and was saved as a pickle file [39]. Multiple values of C and rbf were chosen to perform grid search and it was found that C = 0.5 and rbf = ’auto’ performed the best. The model, when tested with test images, gave a good accuracy. However, it had the following drawbacks:

- Misclassification of few SuperPixels of road as not road and vice versa.
- SuperPixels of the road far away from the camera were misclassified due to the lack of textural features (very small area).

These problems were tackled by using the following techniques:

- Since the number of misclassified SuperPixels are relatively lower, finding the largest contour helps in neglecting the SuperPixels classified as roads that aren’t a part of the road. This also helps to include the SuperPixels misclassified as not roads which are surrounded by a large number of correctly classified SuperPixels.
- Convex hulls can be used to correct the errors due to misclassification of the SuperPixels at the edges of the road.
- The points obtained from the convex hull are then used to predict the edges of the road far away from the camera using regression.

**Fig 7:**
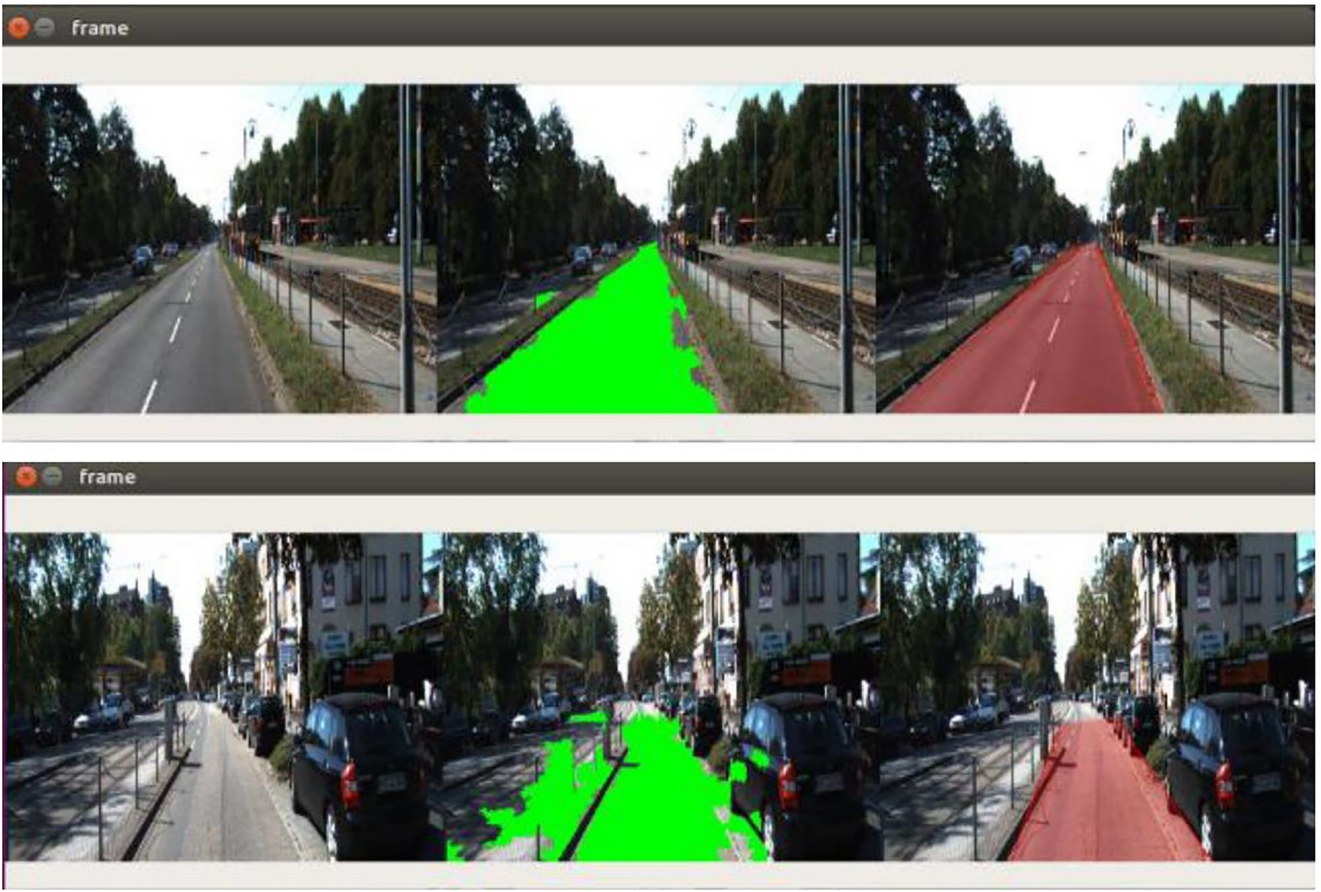
Tackling the obstacles in road perception using different methods to remove the false negatives and consider the false positives.

These problems and their solution can be seen in Fig 7, as shown below, showing the input image, classified SuperPixels, and identified road lane. In general, it was observed that the number of false positives dominate the number of false negatives.

## 5. Limits and discussion

**Fig 8:**
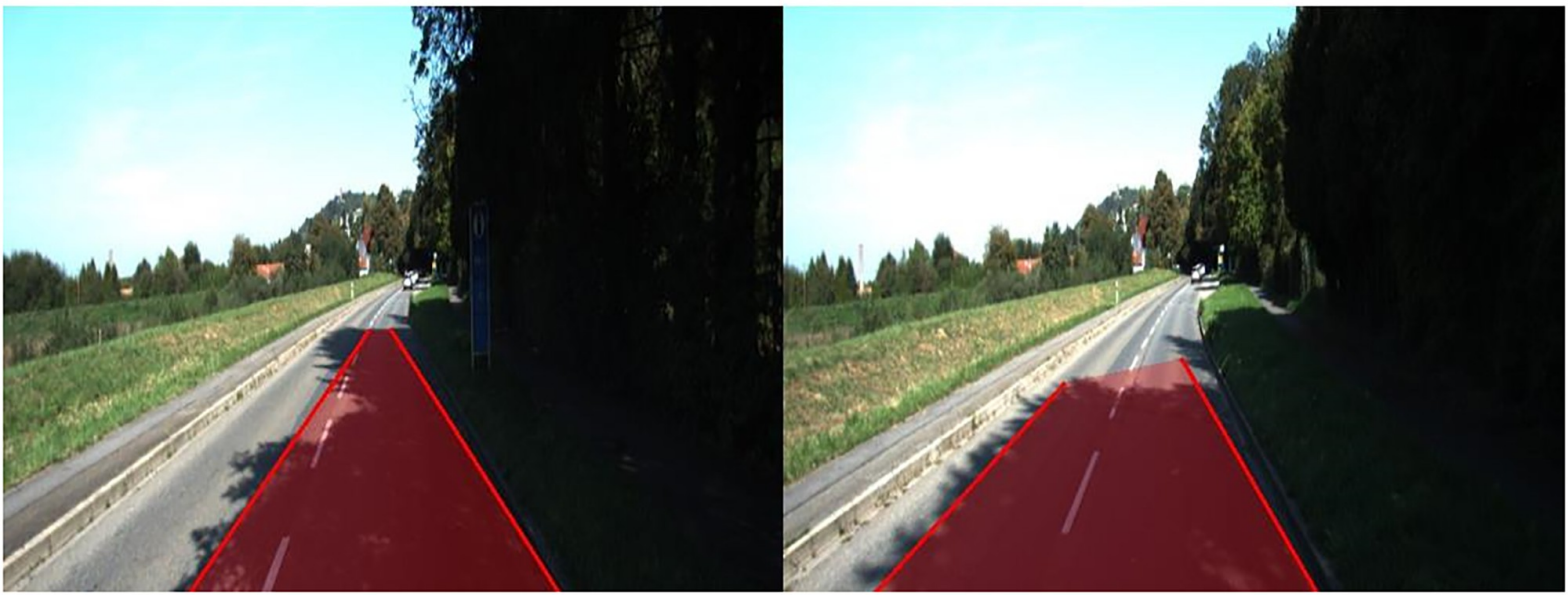
Shadows affecting the color and texture of an input image due to lack of lighting.

**Fig 9:**
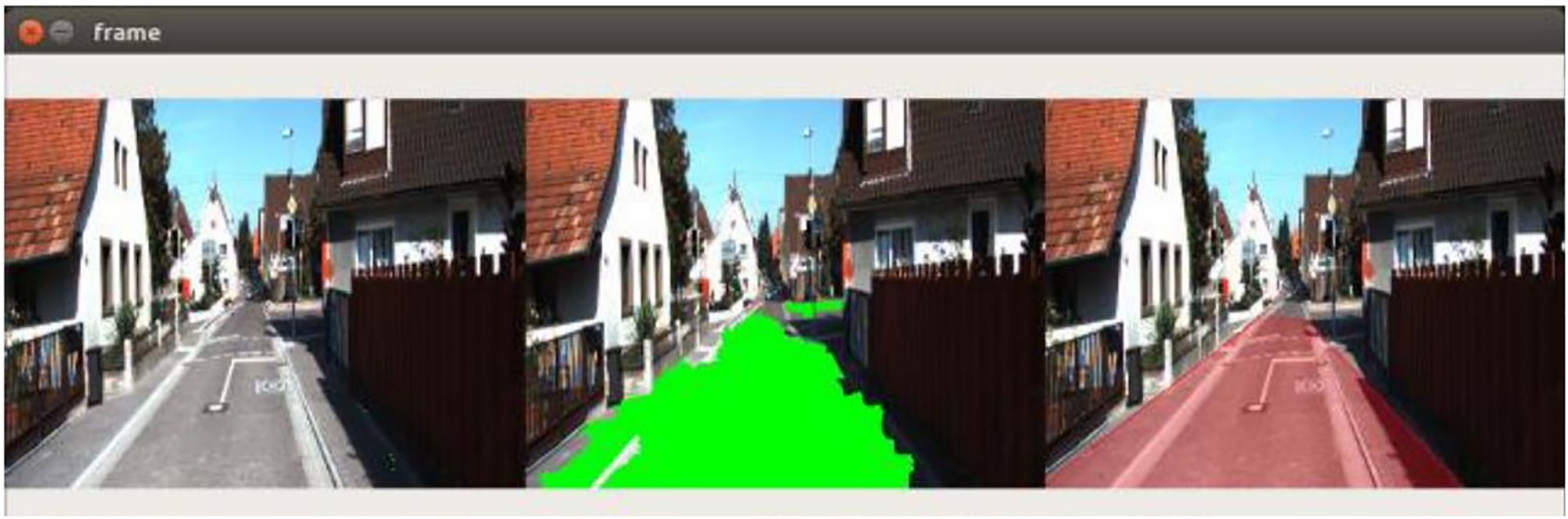
Misclassifying walk paths due to it having the same colour and texture as the road.

The original CycleGAN paper showed that transformations involving geometric changes result in poor performance. They also concluded that more work is required in that area and at the moment, transformations involving textural changes are a suitable application. In this project, we have shown a similar application called sim2real transfer. The road perception task worked very well in real-time producing outputs of more than 30 frames per second while utilizing an Intel core i7 8^th^ gen processor. Some limitations and their possible solutions for road perception are discussed below:

- The presence of large shadows covering a large portion of the road leads to loss of color as well as texture hence misclassifying the entire region as shown in Fig 8. One possible solution is, since this is due to the lack of properties of a 2D image, use of other sensors like LiDAR to sense the texture using depth and fusing the obtained data can be helpful. Data from the previous frames of the camera feed also can be used to identify the position of the shadow.
- Pavements with similar color and textural features of the road can sometimes be misclassified as road. One possible solution is, edge detection along with Hough Transform can be used to identify the intersection of road and pavement. Fig 9 shows this limitation by using a sample image from KITTI and the misclassifications of SuperPixels.
- Junctions where roads merge or diverge, can’t be included within two lines, thus making turns difficult. One possible solution is to use data from GPS and street map to identify junctions and using a different method for those cases.
- Improving the speed of textural feature extraction, which occupies a significant portion of the total processing time. One possible solution is to use DL algorithms like FCNN, hence making it extract the required features by itself during training time.

## 6. Conclusion

In this work, we demonstrated a robust sim2real algorithm that converts the labels of a semantic segmentation map to a realistic-looking street view. The Cityscapes dataset was used to get the semantic segmentation map and then cycle GAN architecture. Further, the generalizability of the CycleGAN architecture was tested by using an origami robot dataset for sim2real transfer. The results shown were found to be qualitatively satisfactory for proposed applications. In addition, road perception was done using a lightweight SVM pipeline and evaluated on the KITTI dataset. The post-processing methods show that the accuracy can be greatly improved without using complex neural network architectures. The Cityscape dataset was used to generate the semantic segmentation map. Then, a novel two-stage generator and discriminator based cycle GAN architecture was introduced to generate synthetic road images, which looks very similar to the original street view images of both Cityscape and KITTI datasets. The SLIC based superpixel extraction method provided a promising result of clustering the pixels within the image with reduced computation time and improved Identity and Cycle consistency loss. The two-stage generator network also learns from the discriminative support vector classifier, which classifies the generated images based on the quality of the synthetic images. This evaluation helps the generator network to fine tune the hyper parameters. The super-pixel classifier helps the discriminator to distinguish between the road and non-road surfaces by 12 parameters for 500 super-pixels per image. This work proposed a hybrid cycle GAN customized with super pixel classifier(in generator network) and SVM classifier (in discriminator network) generates high-quality synthetic images for light weight road perception pipeline.
